# Assessing the Antifungal Effects of Posaconazole and Voriconazole on Soft Liners

**DOI:** 10.7759/cureus.75215

**Published:** 2024-12-06

**Authors:** Madhurima Sharma, Taniya Bhatia, Rohit Sharma, Rohit Nandan

**Affiliations:** 1 Prosthodontics, Teerthanker Mahaveer Dental College and Research Centre, Moradabad, IND; 2 Conservative Dentistry and Endodontics, Teerthanker Mahaveer Dental College and Research Centre, Moradabad, IND

**Keywords:** antifungal, candida albicans, posaconazole, soft liner, voriconazole

## Abstract

Introduction

The cushion effect of soft liners serves to distribute the mastication forces and stresses more evenly, along with absorbing energy. Instead, soft liners can act as a nidus for microbial growth, especially Candida species. An accumulation of these fungi is a problem encountered during the clinical use of them, especially in immunocompromised individuals. This study aimed to evaluate and compare the antifungal activity of soft liners when combined with posaconazole and voriconazole.

Methods

For this study, one soft liner and two antifungal drugs were used. Before testing, drugs were incorporated in a soft liner, and discs were made that were allowed to be immersed in distilled water. Three groups of five samples each were used to categorize the specimens: groups I, II, and III were GC soft liner, GC soft liner + posaconazole, and GC soft liner + voriconazole, respectively. These samples were assessed for antifungal activity on the 1st, 10th, and 20th day. For antifungal activity, the zone of growth inhibition was measured.

Results

A statistically significant difference in zone of growth inhibition across three groups was observed. Group II had the highest zone of inhibition, followed by group III and group I, which had the least. In intragroup comparisons over different time intervals, statistically significant variations were observed; day 20 had the highest zone of inhibition, followed by day 10 and day 1.

Conclusion

Significant changes were seen in the antifungal properties of posaconazole and voriconazole with the soft liner. Posaconazole in soft liners showed a significantly higher zone of growth inhibition than the other two groups.

## Introduction

Fabricating a removable complete denture (RCD) for a totally edentulous patient is typically regarded as a difficult technique. The effectiveness of delivering a functional prosthesis is heavily dependent on the practitioner's expertise and understanding. Patients successfully want their dentist to provide a comfortable denture-wearing experience. However, elderly individuals, who generally have thin and fragile mucosa on their edentulous ridges, confront unique obstacles when wearing dentures. Soft liners, or tissue conditioners (TCs), that serve as cushioning materials beneath these dentures were first utilized in 1869 by a person named Twitchell, offering patients maximum ease in their prosthesis [1-5].

The cushion effect of soft liners serves to distribute the mastication forces and stresses more evenly, along with absorbing energy. Instead, soft liners can act as a nidus for microbial growth, especially Candida species. An accumulation of these fungi is a problem encountered during the clinical use of them, especially in immunocompromised individuals. The fungi coverage increases continuously with the duration of the soft liner retained in the oral cavity. As a result, having a soft liner with an anti-fungal effect is crucial for limiting or reducing such opportunistic infections [6].

*Candida albicans*, an opportunistic fungal infection, is the primary cause of denture-induced stomatitis. At least 65% of older denture wearers have Candida, and these yeasts have been found in 93% of individuals with denture stomatitis (DS). There is data suggesting that Candida can bind to TCs. This initial adhesion is seen as a critical phase that may precipitate the beginning of infection, eventually leading to varying degrees of DS affecting the surrounding mucosa [7].

The treatment of Candida-associated DS is complex due to its multifactorial etiology. The therapeutic strategy adopted ranges from meticulous denture cleaning to the use of systemic as well as topical antifungal agents. Poor response to topical antifungal drugs is common, due to the diluent effect of saliva, swallowing, and tongue movements. Multiple topical applications are required; hence, patient compliance is important. The widespread use of systemic medications has resulted in toxicity, drug interactions, and the growth of resistant species [7].

The main assets of using fungicidal agents in soft liners for drug transfer are as follows: lower costs because a smaller percentage of the fungicidal agent is used than in traditional therapy, no patient acceptance required, treating injured tissue that supports the denture and Candida infection at the same time, and fewer application frequencies [8].

Combining the fungicidal properties of a fungicidal drug with the curative advantages of soft liners was the idea put forth by Douglas and Walker in 1973. This strategy provided sustained pharmacological action, was economical, and encouraged the healing of injured tissue. It could be speculated that the incorporation of a fungicidal agent in a long-term denture liner may be beneficial [9].

## Materials and methods

This study was done in the Department of Prosthodontics and Crown and Bridge at Teerthanker Mahaveer Dental College and Research Centre, Moradabad, India. The study got approval from the Institutional Ethics Committee, Teerthanker Mahaveer Dental College and Research Centre (TMDCRC/IEC/21-22/PCB4).

A stainless-steel metal die of 25 mm diameter and 2 mm thickness as per the American Dental Association (ADA) specification no. 19 was used to fabricate sample discs. GC soft liner was mixed according to the manufacturer’s recommendation (2.2 g/1.8 g) in a mixing jar. The antifungal drugs posaconazole and voriconazole (200 mg of each drug) were crushed using a mortar pestle and added to the polymer in a specified ratio (10% w/w). After completing the process, five samples each of GC soft, GC soft with posaconazole, and GC soft with voriconazole were made.

The finished samples were stored in distilled water in separate labeled containers at room temperature at 37°C for 20 days. At intervals of day 1, 10, and 20 samples were checked for antifungal efficacy. To measure antifungal efficacy, a zone of inhibition was measured.

**Figure 1:**
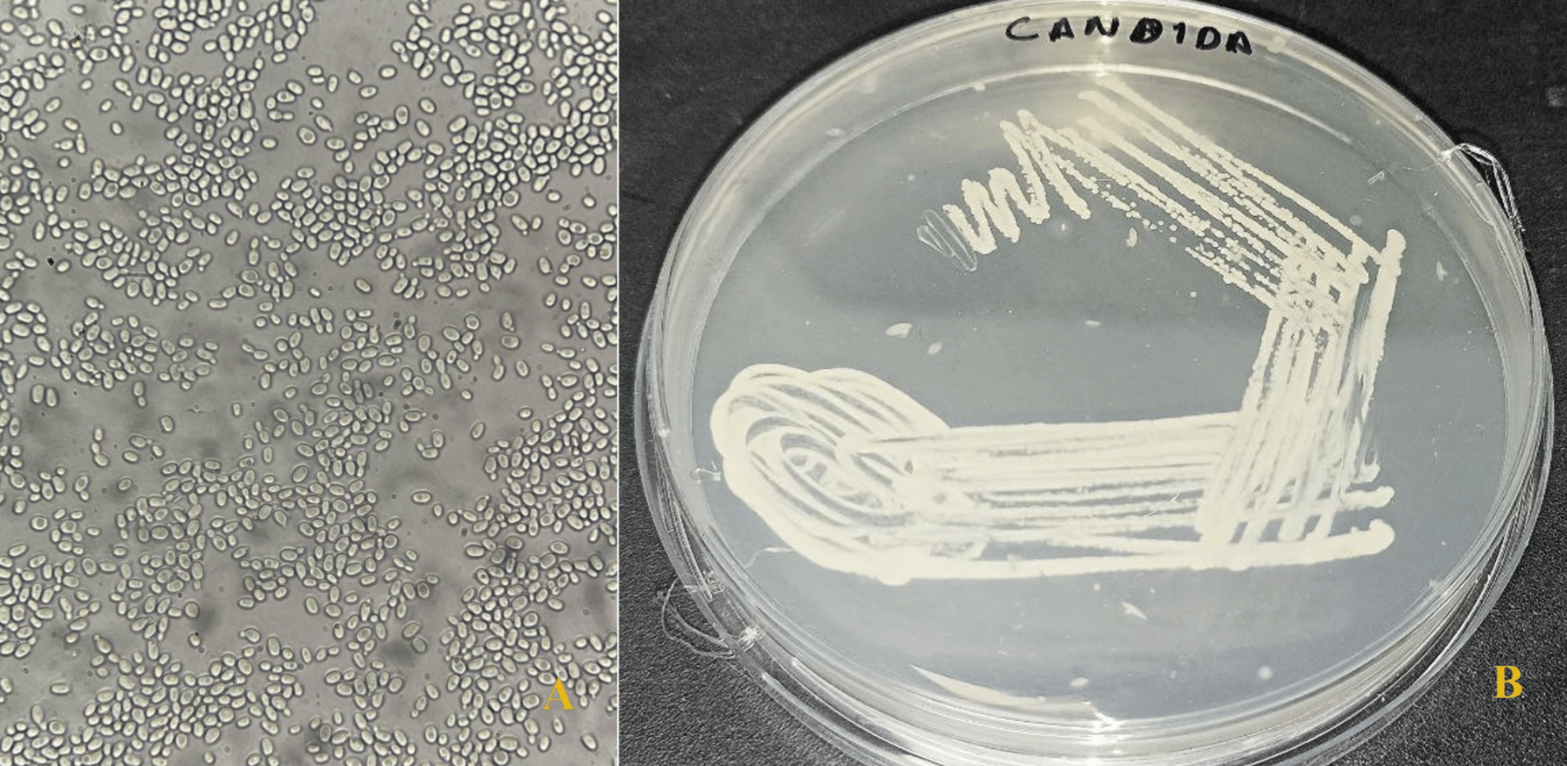
Candida albicans growth A) *Candida albicans* under microscopic level; B) Growth of Candida on SDA plate SDA: Sabouraud dextrose agar

About 1 L of pure, deionized water was utilized to dissolve 61.36 grams of Sabouraud dextrose agar (SDA). It underwent autoclave sterilization. Approximately 25 mL of SDA were added to a petri dish after the mixture had cooled to 40°C. A culture of *C. albicans* was attained from the Ultimate Path Laboratory, Moradabad, India (Figure 1A). SDA petri plates received an inoculation with that Candida inoculum following solidification. The incubation process lasted for 24 hours at 37°C (Figure 1B).

**Figure 2:**
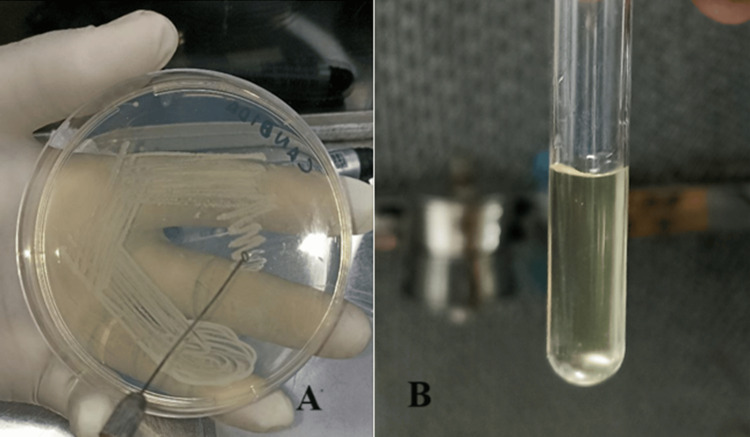
Making of suspension A) Few colonies from the SDA plate were picked up using a sterile inoculum loop; B) Mixing of colonies with 5 ml sterile saline to obtain suspension SDA: Sabouraud dextrose agar

After the intended growth was obtained on a petri plate, few colonies were picked up using a sterile inoculation loop (Figure 2A). These colonies were suspended in 5 ml of sterile saline (0.9%) to obtain suspension. The resulting suspension was centrifuged, and turbidity was set at 0.5 McFarland standard (Figure 2B).

**Figure 3:**
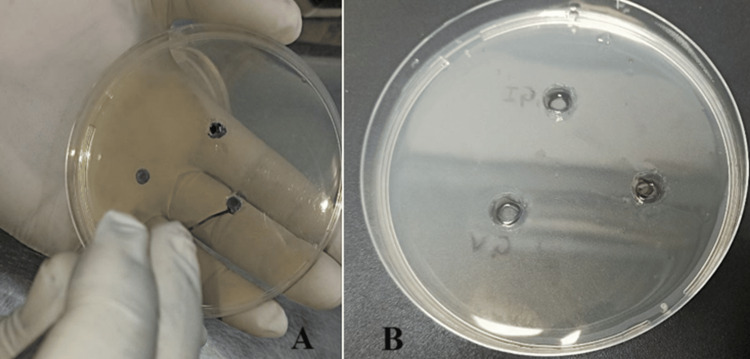
Well formation A) Three wells of 4 mm diameter were made; B) Eluates of each group were added to each well

This suspension obtained was then evenly divided into plates and allowed to solidify, after which three wells, each 4 mm in diameter, were cut in the agar (Figure 3A). The eluates from every test group were placed into each well, and naming was done (Figure 3B).

This was done at each of the test intervals, i.e., day 1, 10, and 20, for all three groups, and the plates were incubated at 37°C for 24 hours to check for the zone of growth inhibition of *C. albicans*.

Statistical analysis

The data for the present study was entered in Microsoft Excel 2007 (Microsoft Corporation, Redmond, USA) and analyzed using IBM SPSS Statistics for Windows, Version 23 (Released 2015; IBM Corp., Armonk, New York, USA). The descriptive figures included mean and standard deviation. The level of significance for the present study was fixed at 5%. The intergroup comparison will be done using the one-way ANOVA test followed by post-hoc analysis depending upon the normality of the data. The Shapiro-Wilk test was used to investigate the allocation of the data and Levene’s test to explore the homogeneity of the variables.

## Results

Intergroup comparison of the zone of inhibition among three groups on day 1

The mean zone of inhibition on day 1 in different groups: I (GC soft liner) was 2.00, II (GC soft liner + posaconazole) was 6.80, and III (GC soft liner + voriconazole) was 5.20. The zone of inhibition was largest in II and least in I (Table 1). The result of the statistical test revealed that the change among the three groups was statistically substantial (p=0.001).

**Table 1:** Intergroup comparison of the zone of inhibition among three groups on day 1 Group I: GC soft liner; Group II: GC soft liner + posaconazole; Group III: GC soft liner + voriconazole Sig: significant

	Mean	Std. deviation	Std. error	Minimum	Maximum	p-value
Group I	2.00	1.490	0.47140	2.00	6.00	0.001 (Sig)
Group II	6.80	2.547	0.80554	5.00	12.00	
Group III	5.20	2.108	0.66667	4.00	10.00	

Intergroup comparison of the zone of inhibition among three groups on day 10

The mean zone of inhibition on day 10 in different groups: I (GC soft liner) was 3.60, II (GC soft liner + posaconazole) was 15.80, and III (GC soft liner + voriconazole) was 12.60. The zone of inhibition was largest in II and least in I (Table 2). The result of the statistical test revealed that the difference between the three groups was statistically significant (p=0.001).

**Table 2:** Intergroup comparison of the zone of inhibition among three groups on day 10 Group I: GC soft liner; Group II: GC soft liner + posaconazole; Group III: GC soft liner + voriconazole Sig: significant

	Mean	Std. deviation	Std. error	Minimum	Maximum	P-value
Group I	3.60	2.00	0.586	3.00	8.00	0.001 (Sig)
Group II	15.80	1.03	0.321	13.00	20.00	
Group III	12.60	3.04	0.925	8.00	18.00	

Intergroup comparison of the zone of inhibition among three groups on day 20

The mean zone of inhibition on day 20 in group I (GC soft liner) was 6.20, in group II (GC soft liner + posaconazole) was 20.00, and in group III (GC soft liner + voriconazole) was 16.80. The zone of inhibition was largest in group II and least in group I (Table 3). The result of the statistical test revealed that the change among the three groups was statistically substantial (p=0.001).

**Table 3:** Intergroup comparison and post-hoc analysis of the zone of inhibition among three groups on day 20 Group I: GC soft liner; Group II: GC soft liner + posaconazole; Group III: GC soft liner + voriconazole Sig: significant

	Mean	Std. deviation	Std. error	Minimum	Maximum	p-value
Group I	6.20	1.763	0.557	5.00	10.00	0.001 (Sig)
Group II	20.00	3.590	1.135	18.00	28.00	
Group III	16.80	3.155	0.997	18.00	26.00	

Intragroup comparison of the zone of inhibition across different time intervals in three groups

The mean zone of inhibition on day 1 in group I (GC soft liner) was 2.00, in group II (GC soft liner + posaconazole) was 6.80, and in group III (GC soft liner + voriconazole) was 5.20. The mean zone of inhibition on day 10 in group I (GC soft liner) was 3.60, in group II (GC soft liner + posaconazole) was 15.80, and in group III (GC soft liner + voriconazole) was 12.60. The mean zone of inhibition on day 20 in group I (GC soft liner) was 6.20, in group II (GC soft liner + posaconazole) was 20.00, and in group III (GC soft liner + voriconazole) was 16.80 (statistically significant differences were observed within the groups across various time frames, with the largest zone of inhibition observed on day 20, followed by day 10, and the smallest on day 1) (Table 4).

**Table 4:** Intragroup comparison of the zone of inhibition across different time intervals in three groups Group I: GC soft liner; Group II: GC soft liner + posaconazole; Group III: GC soft liner + voriconazole Sig: significant

	Day 1	Day 10	Day 20	p-value
Group I	2.00±1.49	3.60±2.17	6.20±1.76	0.001 (Sig)
Group II	6.80±2.54	15.80±1.33	20.00±3.59	0.001 (Sig)
Group III	5.20±2.10	12.60±3.08	16.80±3.15	0.001 (Sig)

## Discussion

The oral mucosa beneath a denture is impacted by DS, sometimes referred to as chronic atrophic candidiasis, which is an inflammatory disease. It is typified by erythema and occasionally edema of the denture-covered mucosa, frequently accompanied by pain or a burning sensation [10-13]. DS affects 11-67% of those who wear full dentures. Its prevalence is up to 72% among the group of institutionalized population [14-16].

A substantial proportion of people wear RCD encounters from DS. Its etiology is complex. The threat of DS can be significantly elevated by several key factors, including inadequate denture fit, inadequate denture care, and *C. albicans *proliferation on denture surfaces, particularly the tissue in touch with denture-seating surfaces. It is evident that an unhygienic condition is associated with a higher risk of Candida infection [12,17-19]. The threat of DS might be increased by denture materials themselves because of surface roughness, and the hydrophobic nature of denture surfaces may promote the adhesion of bacteria [20,21].

The greatest strategy for preserving denture hygiene is to stop the biofilm from growing on dentures [22]. Although DS is common amongst denture users and is linked to poor denture cleanliness, it appears that only a small percentage of denture users actually clean their prostheses thoroughly [23]. Nevertheless, acceptable denture cleansing by marketable denture cleaners is a harmless and operative method for microbial film removal [24,25]. To assist in managing and avoiding DS relapse, it seems acceptable for denture users to have consistent progression sessions, maintain good hygiene, and possibly have their dentures professionally cleaned on a regular basis. According to Von Fraunhofer and Loewy's review, future strategies for lowering the formation of biofilms may include a protective coat that can inhibit bacterial attachment or altering denture constituents to offer a comparatively anionic surface [20,26].

Single or multiple prosthetic teeth replacements are in high demand in today's society, where socializing is valued and older patients wish to appear younger and have a beautiful smile. There appears to be loosening of the denture bases because bone naturally remodels in response to stress and pressure in the form of occlusal loads and denture bases. TC is a great tool for achieving this. Stabilizing the denture bases with TCs can help mask undesired changes in balanced occlusion caused by ongoing remodeling [27].

In this study, one kind of soft denture liner produced by GC Corporation, called GC soft liner, is used. It is intended to cushion and comfort denture users by absorbing pressure and shock when speaking and chewing. The special silicone-based substance used to create GC soft liner has flexibility and durability, improving denture fit and retention [1]. A salient characteristic of GC soft liner is its enduring softness, which contributes to patient comfort [28].

Because of its multiple causes, treating DS linked with Candida can be challenging. Options for treatment include applying oral antifungal medications as well as thorough denture cleaning. Because they are diluted by saliva, swallowing, and tongue motions, topical antifungals frequently don't perform properly. Patients must adhere to the treatment plan since it is necessary for them to apply it several times. Despite their effectiveness, systemic treatments can cause adverse reactions, drug interactions, and the establishment of resistant strains [7].

Rather than applied topically or systemically, antifungals can be put into the substance as a method of drug delivery to achieve greater results because they release slowly and have long-lasting effects. In the present investigation, posaconazole and voriconazole were added to the GC soft liner, and samples were created to offer a local delivery method.

Newer medications have been introduced to treat Candida infections more effectively to battle resistance to classic antifungal treatments such as nystatin and miconazole. In this study, posaconazole and voriconazole were used as substitutes. These medications show promising results in treating problems linked to Candida and might be more effective against resistant strains.

In the result of this study, GC soft liner showed absolutely no inhibition of *C. albicans*, and posaconazole was a more effective antifungal medication than voriconazole. By comparing the mean zone of inhibition, the antifungal activity of the eluents was assessed. It was found that posaconazole and voriconazole combined with a soft liner produced statistically significant differences in the mean zone of inhibition. During the course of 20 days, the mean zone of inhibition for posaconazole was considerably elevated compared to that of voriconazole, indicating that posaconazole likely exhibited superior anticandidal activity.

There were mathematically substantial disparities between the groups when comparing intragroup comparisons across time periods; day 1 showed the lowest zone of inhibition while day 20 showed the highest. This indicates a gradual increase in antifungal activity over time in all groups, most likely as an outcome of the antifungal compounds' delayed release from the soft liner, which inhibits fungal development for a longer period of time. Overall, the results indicate that posaconazole, in particular, can be added to soft liners to effectively increase their antifungal effectiveness against Candida species. To optimize the formulation and dosage of antifungal drugs for long-term efficacy in dental prostheses, more study is necessary. Overall, the results point to the need for more research to create long-lasting antifungal formulations for use in dental prosthetics.

Limitations of the study

To ascertain the clinical behavior of the included antifungal drugs, the current in vitro study's findings ought to be combined with an in vivo investigation. Prior to utilizing the impregnated soft-liner polymer in clinical settings to treat denture-associated candidiasis, it is imperative to thoroughly evaluate its mechanical and physical characteristics. The zone of inhibition for posaconazole and voriconazole was assessed for 20 days in the current investigation. By examining the long-term local delivery system of both antifungal drugs, it is necessary to analyze the material's durability and serviceability to confirm its clinical performance.

## Conclusions

Within the limitations of this in vitro study, significant changes were seen in the antifungal properties of posaconazole and voriconazole with soft liner. The mean zone of growth inhibition of posaconazole in soft liner was significantly higher than that of voriconazole, thus showing that posaconazole has exhibited significantly better antifungal properties than that of voriconazole.
